# Correlates of wanting to seek help for mental health and substance use concerns by sexual and gender minority young adults during the COVID-19 pandemic: A machine learning analysis

**DOI:** 10.1371/journal.pone.0277438

**Published:** 2022-11-16

**Authors:** Anasua Kundu, Rui Fu, Daniel Grace, Carmen H. Logie, Alex Abramovich, Bruce Baskerville, Christina Yager, Robert Schwartz, Nicholas Mitsakakis, Lynn Planinac, Michael Chaiton

**Affiliations:** 1 Institute of Medical Science, University of Toronto, Toronto, Canada; 2 Centre for Addiction and Mental Health, Toronto, Canada; 3 Ontario Tobacco Research Unit, University of Toronto, Toronto, Canada; 4 Department of Otolaryngology—Head and Neck Surgery, Sunnybrook Research Institute, University of Toronto, Toronto, Canada; 5 Dalla Lana School of Public Health, University of Toronto, Toronto, Canada; 6 Factor-Inwentash Faculty of Social Work, University of Toronto, Toronto, Canada; 7 United Nations University Institute for Water, Environment & Health, Hamilton, Canada; 8 Department of Psychiatry, University of Toronto, Toronto, Canada; 9 Canadian Institutes of Health Research, Ottawa, Canada; 10 School of Pharmacy, Faculty of Science, University of Waterloo, Kitchener, Canada; 11 Children’s Hospital of Eastern Ontario Research Institute, Ottawa, Canada; University Kebangsaan Malaysia, MALAYSIA

## Abstract

The COVID-19 pandemic has worsened the mental health and substance use challenges among many people who are Two Spirit, lesbian, gay, bisexual, transgender, queer, questioning, and intersex (2SLGBTQI+). We aimed to identify the important correlates and their effects on the predicted likelihood of wanting to seek help among 2SLGBTQI+ young adults for mental health or substance use concerns during the pandemic. A cross-sectional survey was conducted in 2020–2021 among 2SLGBTQI+ young adults aged 16–29 living in two Canadian provinces (Ontario and Quebec). Among 1414 participants, 77% (n = 1089) wanted to seek help for their mental health or substance use concerns during the pandemic, out of these, 69.8% (n = 760) reported delay in accessing care. We built a random forest (RF) model to predict the status of wanting to seek help, which achieved moderately high performance with an area under the receiver operating characteristic curve (AUC) of 0.85. The top 10 correlates of wanting to seek help were worsening mental health, age, stigma and discrimination, and adverse childhood experiences. The interactions of adequate housing with certain sexual orientations, gender identities and mental health challenges were found to increase the likelihood of wanting to seek help. We built another RF model for predicting risk of delay in accessing care among participants who wanted to seek help (n = 1089). The model identified a similar set of top 10 correlates of delay in accessing care but lacked adequate performance (AUC 0.61). These findings can direct future research and targeted prevention measures to reduce health disparities for 2SLGBTQI+ young adults.

## 1. Introduction

Two Spirit, lesbian, gay, bisexual, transgender, queer, questioning, and intersex (2SLGBTQI+) young adults experience significant mental health disparities and substance use challenges [1–5]. In 2019, 46.8%% of lesbian, gay or bisexual (LGB) youth in the United States (US) reported having serious thoughts of attempting suicide compared to only 14.5% of their heterosexual peers. Similarly, the rate of illicit drug use was reported to be more than twice among the LGB youth (27.8% among LGB vs 12.7% among heterosexual) [4]. Recent meta-analysis found that 2SLGBTQI+ youth had 2.67 times higher odds of experiencing mental health difficulties than heterosexual and cisgender counterparts [3]. Moreover, lifetime use of substances was reported 1.48 times more likely among transgender individuals compared to the cisgender people [5].

Growing evidence has demonstrated that that the recent pandemic outbreak of Coronavirus disease 2019 (COVID-19) exacerbated the mental health and substance use problems among sexual and gender minority (SGM) people [6–8]. A Canadian prospective study showed that SGM participants had experienced higher and increasing rate of deterioration of mental health, suicidal thoughts, alcohol, cannabis and substance use compared to non-SGM people [6]. Moreover, multiple reports of difficulty in accessing healthcare by 2SLGBTQI+ people is concerning and needs urgent attention [7,9]. However, it is not clear which individual-level factors may have contributed to the desire to seek help for mental health and substance use concerns among 2SLGBTQI+ young adults during the pandemic. These results have crucial implications to guide policy interventions that target this specific population. We aimed to identify these factors by using machine learning analysis—a novel statistical method which has emerged as a promising means of analyzing a vast range of complex data in public health informatics [10,11]. Our recent scoping review of machine learning applications in mental health and substance use issues among SGM people found only 4 prediction modelling studies up to 2020 [12]. We recently updated the review and detected 3 additional studies [13–15] that applied machine learning-based predictive modelling and none of them explored the wanting to seek help behaviours for mental health and substance use issues among this population. Hence, we addressed this research gap by this current study.

Machine learning has also been increasingly used as an important tool in the field of intersectional research of inequalities targeting socio-demographic identities and social positions (e.g., age, sex, gender, ethnicity, socio-economic status) [16,17] through providing newer techniques and methods [18,19]. Recent data suggests that 84% and 66% of Canadian homeless 2SLGBTQI+ young adults experienced severe anxiety and moderately severe depression respectively, while 57% of these young adults reported problematic substance use during the pandemic [20]. Moreover, unemployment and urbanicity have been found to increase the risk of suicidal thoughts among SGM youth who experienced mental health challenges or social stigma [13]. It is important to understand how these interactions of SGM status with different socio-economic identities influence needs of seeking help. In addition, as 2SLGBTQI+ people encounter stigma, bullying, violence and institutional discrimination throughout their lifespan [21–23], further contributing to the development of mental health challenges and substance use concerns among them [3,23,24]. Hence, understanding the role of these risk factors behind wanting to seek help and accessing care could help policy makers to plan equitable distribution of healthcare.

The primary objective of our study was to identify the important correlates and their effects on the predicted risk of wanting to seek help for mental health and substance use concerns among 2SLGBTQI+ young adults during the pandemic. We also aimed to assess the prevalence and correlates of delay in accessing care among 2SLGBTQI+ young adults who wanted to seek help from health professionals as a secondary objective.

## 2. Materials and methods

### 2.1 Data source

Data for this cross-sectional analysis were taken from the 2SLGBTQI+ Tobacco Project Survey, conducted among 2SLGBTQI+ young adults aged 16–29 years living in Canada during December 2020 to March 2021. This study was approved by the Centre for Addiction and Mental Health Research Ethics Board and adhered to the principles of the Declaration of Helsinki. Individuals provided written informed consent before participating in this study. Participants were recruited from two Canadian provinces: Ontario and Quebec. We received 1511 responses and participants were compensated $10 CAD. Participants who did not complete the survey were excluded, resulting in a total of 1414 respondents.

All statistical analyses were performed using R, version 4.0.5 (R Foundation for Statistical Computing). The survey questionnaire and the R codes required to reproduce the analysis are available on the Open Science Framework (https://osf.io/tnd2u/) and the deidentified data can be accessed from the University of Toronto Dataverse [25]. The entire analytical framework of the machine learning models from data pre-processing to sensitivity analysis were presented in S1 Appendix.

### 2.2 Outcome measures

We assessed two binary outcomes in this analysis, including a primary outcome defined for the entire study sample and a secondary outcome defined only for a subgroup of participants. Our primary outcome was the status of wanting to seek help from health professionals for mental health or substance use concerns during the pandemic, which was denoted as March 2020 [26,27]. This variable was defined using the survey question, ‘Since March 2020, was there a time when you wanted to talk with or seek help from a health professional about stress, depression, problems with emotions or substance use?’ Next, amongst participants who answered ‘yes’ to this question, i.e., those who had ever wanted to seek help during the pandemic (n = 1089, 77.0% of all participants), we created another binary outcome variable of ‘delay in accessing care’ based on the question ‘Did you delay or not get the care you thought you needed?’

### 2.3 Features

For both outcomes, we considered the same set of 61 features, including 53 categorical and 8 continuous variables (see https://osf.io/tnd2u/ for full list of variables). From the survey, we extracted socio-demographic characteristics (age, sexual orientation, gender, ethnicity, education, employment, income, duration of living in Canada, and housing status); health status (self-rated general and mental health, comorbidity, disability, stress, diagnosis of different mental illness, past year suicidal thoughts and past-week depression score); frequency of smoking, vaping, other tobacco products use, alcohol drinking, cannabis and other drug use; and perceived COVID-19 impact on smoking and other substance use. Additionally, we created several composite score variables based on validated scales measures, which included overall scores of enacted and perceived stigma [28], internalized homophobia [29], identity centrality [30], community connectedness [31], average scores of outness [32] and overall scores of adverse childhood experiences (ACEs) [33].

### 2.4 Descriptive analysis and data pre-processing

We summarized characteristics of the participants based on the status of wanting to seek help (n = 1414) and delay in accessing care (n = 1089), respectively. For continuous variables, we used 2-sample t-test and for categorical variables, we used Fisher’s Exact Test to compare the characteristics in both descriptive analyses. Due to the presence of small cell counts (n<5), we used Fisher’s Exact Test instead of Pearson Chi-square test to robustly identify any significant difference between groups [34].

We performed standard data preprocessing procedures to create a dataset ready for the machine learning analysis. We removed or recoded low-variance variables, defined as those with percentage of unique values <5% [35,36]. Inspection was carried out for all variables to rule out collinearity (i.e., all pairwise Cramér’s V<0.6) [37]. For missing data found in our dataset (2.8% total missingness with 15 variables showing ≥5% missing data), we handled them using the Multivariate Imputation by Chained Equations (MICE) approach [38] after confirming the assumption of missing at random was plausible. Specifically, 5 MICE-imputed datasets were independently generated, where based on the performance (highest AUC) of the models, one dataset was chosen to be used in the primary analysis, with the remainder being assessed in the sensitivity analysis. Before running the final model, we created the composite score variables in the imputed data. Notably, out of 1414 participants, 11% (n = 161) declined to respond to the ACEs questionnaire, which were managed by MICE imputation before creating overall scores of ACEs. No feature selection was performed due to the relatively lower number of total variables (61 variables). We additionally used ‘missForest’, a random forest-based iterative algorithm [39] to examine the impact of missing data in the sensitivity analysis.

### 2.5 Random forest analysis

We performed separate random forest (RF) analysis for the primary outcome (on the entire study sample, n = 1,414) and the secondary outcome (on those who had wanted to seek help, n = 1,089), following the same analytical pipeline (S1 Appendix). We specifically chose RF due to its proven effectiveness in public health applications, particularly in predicting smoking and vaping outcomes, and provides higher level of accuracy compared to others [40–42]. In each analysis, we randomly split the imputed dataset at 7:3 ratio to obtain a larger training set for model development and a smaller testing set for evaluating model performance. Using the R package ‘caret’ [43] and 10-fold cross-validation method [44], we built the RF model on the training dataset using the default setting for hyperparameters (S1 Appendix). Performance of the RF model was evaluated on the testing set using the receiver operating characteristic (ROC) curve. The area under the ROC curve (AUC) was computed to give an overall account of the classification ability of the model while high performance was declared using an AUC threshold of 0.80 [45]. Accuracy, sensitivity, and specificity of the RF models were also calculated. If the final model failed to reach a testing AUC of 0.70 [46], we implemented regularized logistic regression using the Lasso (least absolute shrinkage and selection operator) [47] to see if this would yield a better performance (see sensitivity analysis).

### 2.6 Interpreting the random forest findings

For each outcome, we used the respective RF model to conduct *post-hoc* analyses to provide interpretable policy-relevant findings. First, we identified the top ten most important contributing features (i.e., correlates) based on a relative importance score calculated for each variable. These scores corresponded to the loss of prediction accuracy of the RF model due to the exclusion of a variable [48,49]. We ranked all the variables on a scale of 0% to 100% with the most important one receiving the highest score of 100%. For our primary outcome (wanting to seek help), we additionally illustrated the one-way partial dependence plots (PDPs) for each of its top ten correlates to demonstrate their marginal effect. We further assessed the partial dependence based 2-way interaction effects [50] by identifying pairs of variables that were jointly significant in predicting the probability of wanting to seek help. This was done by examining the statistical strength of all 91 pairs of interactions formed by sociodemographic variables (such as age*gender) and/or the previously identified top 10 correlates, following the procedures conducted in a recent machine learning paper [51]. We were particularly interested in studying interactions between sociodemographic variables to demonstrate the effect of intersectionality [52]. Two-way PDPs were then used to demonstrate the top 10 strong interactions and their marginal effects.

### 2.7 Sensitivity analysis

Sensitivity analyses were conducted to examine the robustness of the primary findings. First, we repeated the analyses separately on the remaining 4 MICE imputed datasets. Second, we also ran completed-case analyses by only including participants with full data (n = 364, 25.7% of all participants for the primary outcome; n = 293, 26.9% of all participants for the secondary outcome), although these models were expected to have poor performance. Third, to further assess the impact of missing data, we implemented the ‘missForest’ algorithm and compared the resulted RF models with our primary models. We used ‘missForest’ because it was found to outperform other data imputation algorithm including MICE and *K*-nearest neighbour imputation [39]. Fourth, we derived a logistic regression model using lasso if the primary RF model failed to reach an AUC of 0.70. The lasso method achieves a sparse model by penalizing the number of predictors and only retaining one of the two correlated predictors [47].

## 3. Results

### 3.1 Sample characteristics

Among the 1414 2SLGBTQI+ participants aged 16–29 years, 77.0% (n = 1089) wanted to seek help during the pandemic, while 19.9% (n = 282) participants reported not wanting to seek any help (Table 1). Nearly all (n = 1372, 97.0%) participants lived in urban areas and had an average age of 21.9 years (standard deviation [SD] = 3.78). Participants identified themselves with a range of gender identities, 45.1% (n = 607) of participants identified as cisgender women, 16.3% (n = 220) as cisgender men, 6.9% (n = 93) as transgender women, 1.8% (n = 24) as transgender men, 29.9% (n = 402) as gender diverse including genderqueer, non-conforming, non-binary, or Two-Spirit, and 2.0% (n = 26) reported being intersex. Among the sexual orientation categories, 29.8% (n = 421) identified themselves as bisexual, 16.5% (n = 233) as lesbian, 15.9% (n = 225) as gay, 17.2% (n = 243) as queer, 1.6% (n = 22) as questioning, and 14% (n = 198) as other. Most (68.2%; n = 922) of the total participants were White and 31.8% (n = 430) were persons of colour (POC) from different ethnicities such as Asian, African American, Latin American, Middle eastern, Indigenous, and mixed heritage.

**Table 1:** Socio-demographic characteristics of participants by wanting to seek help for mental health or substance use concerns during the pandemic (n = 1414).

Characteristics	Did not want to seek help (n = 282)^a^	Wanted to seek help (n = 1089)^b^	Total (n = 1414)^c^	p-value
**Age (years), mean (SD)**	22.54 (3.71)	21.75 (3.78)	21.90 (3.78)	0.002
**Sexual orientation, n (%)**	** **			<0.001
Asexual	14 (21.2)	50 (75.8)	66 (4.7)	
Straight or heterosexual	1 (16.7)	5 (83.3)	6 (0.4)	
Bisexual	66 (15.7)	339 (80.5)	421 (29.8)	
Gay	91 (40.4)	126 (56.0)	225 (15.9)	
Lesbian	57 (24.5)	169 (72.5)	233 (16.5)	
Queer	26 (10.7)	214 (88.1)	243 (17.2)	
Questioning	7 (31.8)	14 (63.6)	22 (1.6)	
Others	20 (10.1)	172 (86.9)	198 (14.0)	
**Gender identity, n (%)**				<0.001
Cisgender man	100 (45.5)	116 (52.7)	220 (16.3)	
Cisgender woman	112 (18.5)	479 (78.9)	607 (45.1)	
Transgender man	10 (10.8)	79 (84.9)	93 (6.9)	
Transgender woman	6 (25.0)	17 (70.8)	24 (1.8)	
Gender diverse	44 (10.9)	344 (85.6)	402 (29.9)	
**Intersex, n (%)**	6 (23.1)	17 (65.4)	26 (2.0)	0.756
**Ethnicity, n (%)**				0.724
White	180 (19.5)	718 (77.9)	922 (68.2)	
Person of colour	87 (20.2)	326 (75.8)	430 (31.8)	
**Education, n (%)**				<0.001
High school or below	66 (15.9)	333 (80.4)	414 (29.7)	
College or diploma	83 (16.4)	408 (80.8)	505 (36.3)	
University degree	126 (26.6)	337 (71.2)	473 (34.0)	
**Employment, n (%)**				<0.001
Unemployed	144 (14.9)	792 (82.1)	965 (68.9)	
Employed	131 (30.0)	293 (67.2)	436 (31.1)	
**Household income, n (%)**				<0.001
Yearly less than $60,000	79 (14.3)	458 (83.1)	551 (51.7)	
Yearly $60,000- $100,000	107 (35.9)	187 (62.8)	298 (28.0)	
Yearly more than $100,000	39 (18.1)	170 (78.7)	216 (20.3)	
**Type of municipality, n (%)**				0.448
Rural	6 (14.3)	35 (83.3)	42 (3.0)	
Urban	276 (20.1)	1054 (76.8)	1372 (97.0)	
**Housing, n (%)**				0.408
Inadequate	32 (22.2)	105 (72.9)	144 (10.3)	
Adequate	244 (19.4)	979 (77.9)	1256 (89.7)	
**Substance use in past 12 months, n (%)**	66 (15.2)	356 (82.0)	434 (31.1)	0.003
**Anxiety disorder, n (%)**	41 (6.6)	561 (90.3)	621 (43.9)	<0.001
**Depression, n (%)**	35 (6.6)	489 (91.6)	534 (37.8)	<0.001
**General health, n (%)**				<0.001
Poor or fair	57 (10.5)	471 (87.1)	541 (38.9)	
Good or excellent	221 (26.0)	603 (71.0)	849 (61.1)	
**Mental health, n (%)**				<0.001
Poor or fair	93 (9.6)	851 (88.2)	965 (68.7)	
Good or excellent	188 (42.7)	234 (53.2)	440 (31.3)	
**Current stress level, n (%)**				<0.001
Not stressful or neutral	185 (34.1)	336 (61.9)	543 (38.4)	
Very stressful or extremely stressful	97 (11.1)	753 (86.5)	871 (61.6)	
**COVID-19 impact on smoking, n (%)**				<0.001
Don’t smoke	150 (16.5)	739 (81.4)	908 (64.2)	
Increased	46 (25.0)	130 (70.7)	184 (13.0)	
Decreased	17 (15.3)	87 (78.4)	111 (7.9)	
No change	65 (38.7)	94 (56.0)	168 (11.9)	
Quit	4 (9.3)	39 (90.7)	43 (3.0)	
**COVID-19 impact on alcohol and substance use, n (%)**				<0.001
Don’t use	100 (35.1)	169 (59.3)	285 (20.2)	
Increased	54 (10.9)	428 (86.5)	495 (35.0)	
Decreased	51 (19.7)	204 (78.8)	259 (18.3)	
No change	60 (20.4)	225 (76.5)	294 (20.8)	
Quit	17 (21.0)	63 (77.8)	81 (5.7)	

Abbreviations: COVID-19, Coronavirus disease 2019; SD, Standard deviations.

^a^ The percentage in the brackets represents rate of the participants in each characteristic who did not want to seek help.

^b^ The percentage in the brackets represents rate of the participants in each characteristic who wanted to seek help.

^c^ The percentage in the brackets represents percentage of the total participants in each characteristic.

Note: We used 2-sample t-test and Fisher’s Exact Test to compare the distribution of continuous and categorical variables between the two groups.

Wanting to seek help was found to differ significantly by age, sexual orientation and gender identity, education, employment, and household income (p<0.01). Notably, those who wanted to seek help were slightly younger on average (mean age 21.75 ± 3.78 years). More than 80.0% of the participants with bisexual (n = 339), queer (n = 214) and others (n = 172) sexual orientation wanted to seek help. Approximately 85.0% of the transgender men (n = 79) and gender diverse (n = 344) participants as well as almost 80.0% of the cisgender women (n = 479) wanted to seek help. The rate of wanting to seek help was 80.6% (n = 741) among participants who had less than a university degree, 82.1% (n = 792) among unemployed, 83.1% (n = 458) among those who had a past year household income of less than $60,000. In addition, wanting to seek help was also significantly (p<0.01) higher among past year substance users (82%, n = 356), those with anxiety disorder (90.3%, n = 561) and depression (91.6%, n = 489), higher stress levels (86.5%, n = 753), poor or fair mental health (88.2%, n = 851) and general health (87.1%, n = 471), and who reported quitting smoking (90.7%, n = 39) or increased alcohol drinking or other substance use (86.5%, n = 428) as impacts of COVID-19.

### 3.2 Performance of the RF model for wanting to seek help

**Fig 1:**
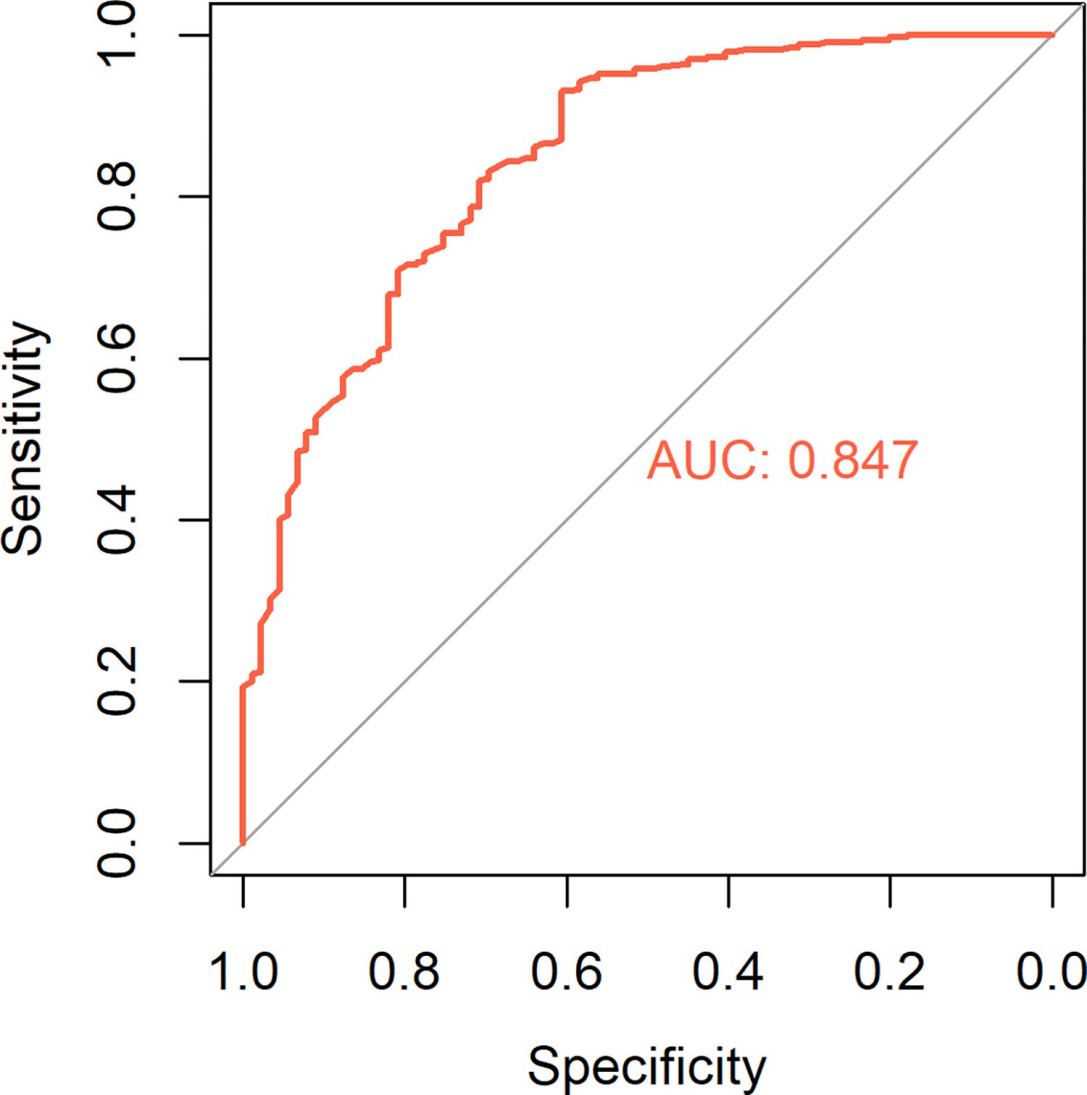
The receiving operating characteristic curve of the random forest on the testing set for predicting wanting to seek help.

The RF model built for predicting the status of wanting to seek help for mental health or substance use issues showed moderately high performance, with accuracy 0.86 (95% CI 0.83, 0.89), sensitivity 0.53 (95% CI 0.42, 0.63), specificity 0.95 (95% CI 0.92, 0.97) and AUC 0.85 (Fig 1).

### 3.3 Top 10 correlates of wanting to seek help

**Fig 2:**
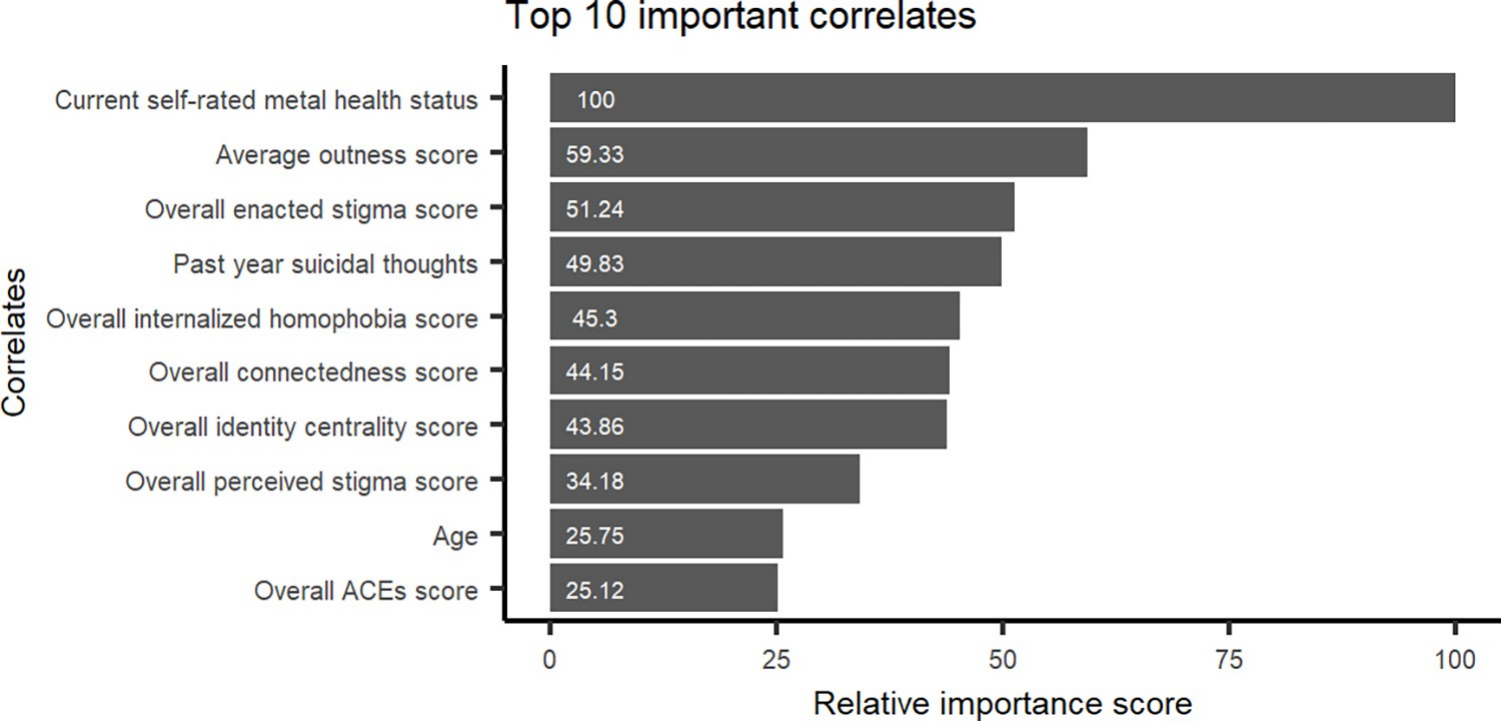
The top ten correlates of wanting to seek help and their scaled relative importance.

**Fig 3:**
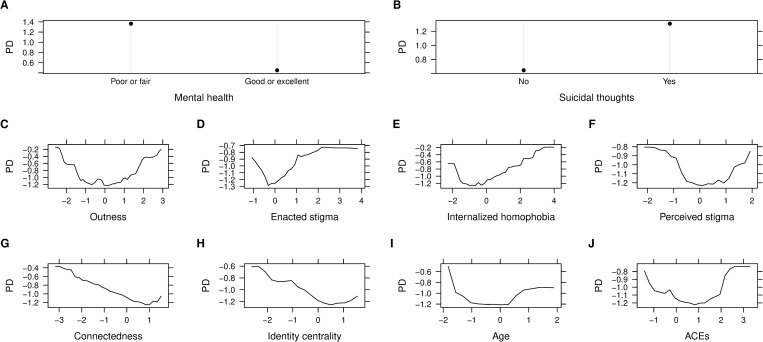
Partial dependence of wanting to seek help on top 10 important correlates. A) Current self-rated mental health status; B) Past year suicidal thoughts; C) Average outness score; D) Overall enacted stigma score; E) Overall internalized homophobia score; F) Overall perceived stigma score; G) Overall connectedness score; H) Overall identity centrality score; I) Age; J) Overall adverse childhood experiences (ACEs) score. Note: Numeric variables (average outness score and overall internalized homophobia score) were standardized before model training.

The top 10 correlates of wanting to seek help identified from the RF model were (Fig 2): current self-rated mental health status (100%), average outness score (59.3%), overall enacted stigma score (51.2%), past-year suicidal thoughts (49.8%), overall internalized homophobia score (45.3%), overall connectedness score (44.2%), overall identity centrality score (43.9%), overall perceived stigma score (34.2%), age (25.8%), and overall ACEs score (25.1%). One-way partial dependence plots (PDPs) depicting the marginal effects of the top 10 correlates are presented in Fig 3. Overall, the likelihood of wanting to seek help was higher among those who rated their mental health as poor or fair, reported to have suicidal thoughts in the past year, higher enacted stigma, higher internalized homophobia, lower connectedness with the community, lower level of identity centrality, and those who rated their outness and perceived stigma much higher or much lower than average. The probability of wanting to seek help was highest among the youngest age groups, and also higher among older age groups. Participants with very high ACEs score showed the highest probability of wanting to seek help, while the probability was also quite high for those with very low ACEs score.

### 3.4 Interaction effects on predicted risk of wanting to seek help

**Fig 4:**
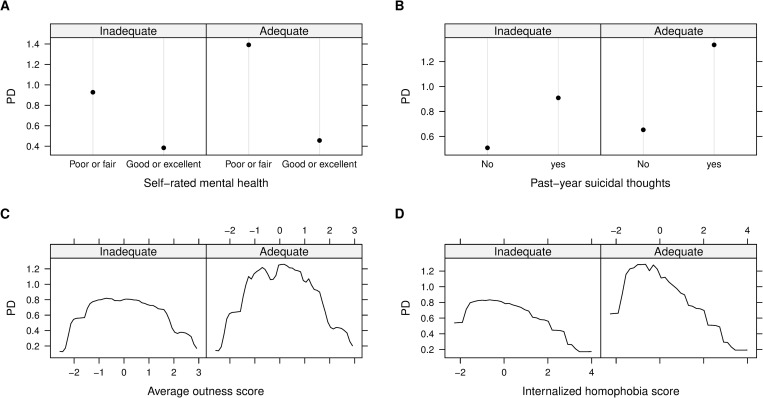
Two-way partial dependence (PD) plots of strong interactions of wanting to seek help. A) current self-rated mental health and housing status; B) past-year suicidal thoughts and housing status; C) Average outness score and housing status; D) Overall internalized homophobia score and housing status. Note: Numeric variables (average outness score and overall internalized homophobia score) were standardized before model training.

**Fig 5:**
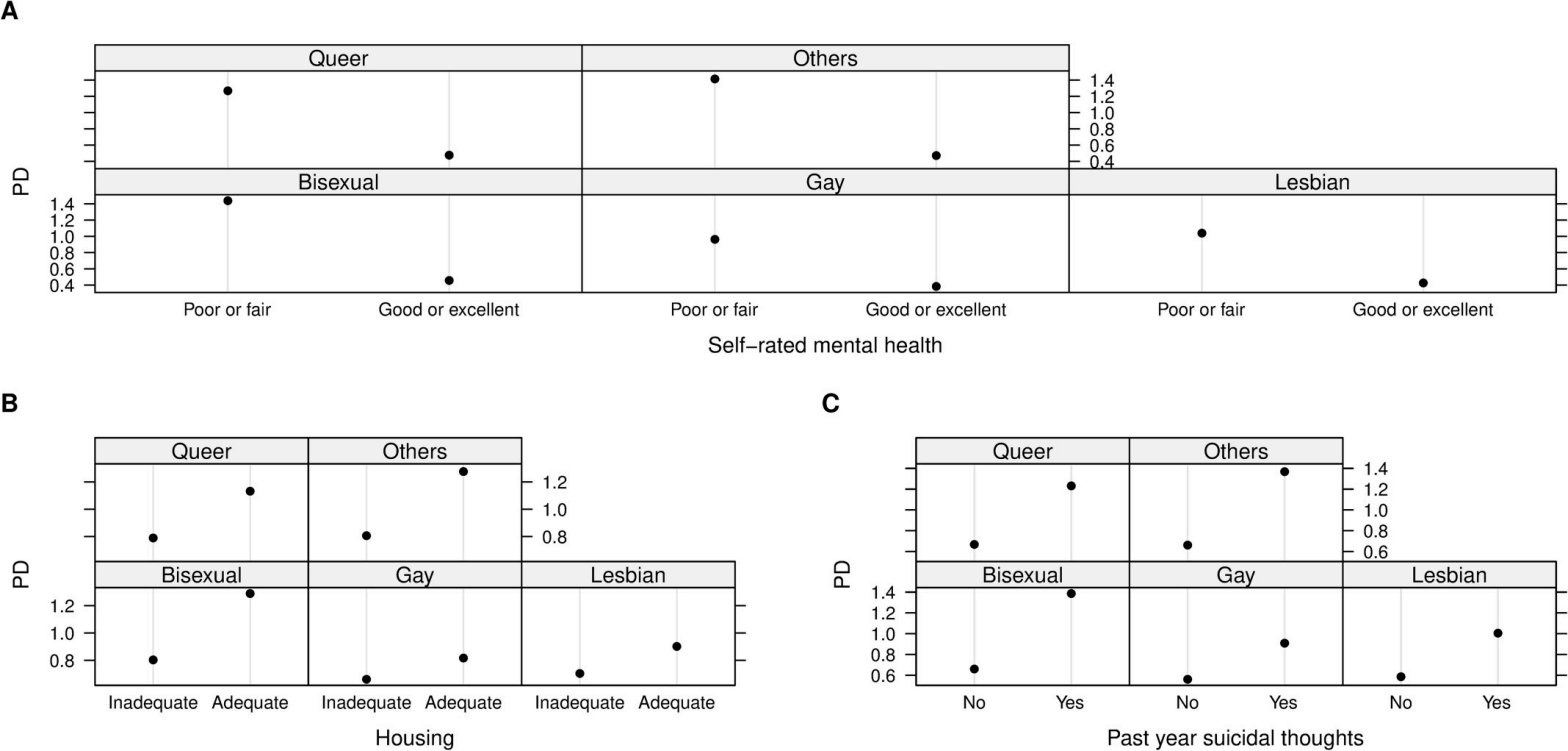
Two-way partial dependence (PD) plots of strong interactions of wanting to seek help. A) current sexual orientation and self-rated mental health; B) current sexual orientation and housing status; C) current sexual orientation and past-year suicidal thoughts.

**Fig 6:**
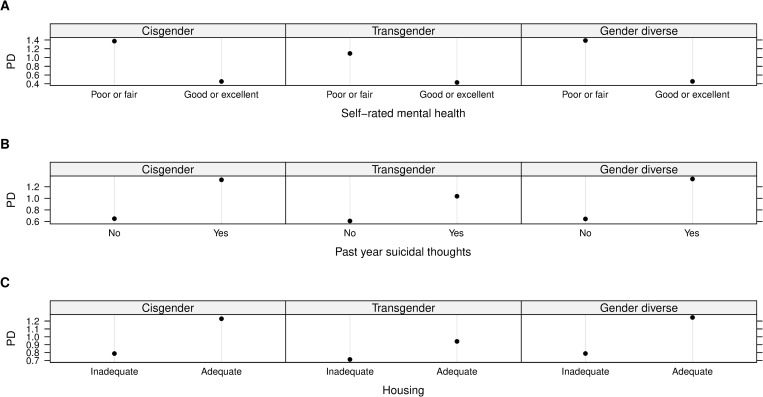
Two-way partial dependence (PD) plots of strong interactions of wanting to seek help. A) gender identity and self-rated mental health; B) gender identity and past-year suicidal thoughts; C) gender identity and housing status.

All pairwise interactions formed by socio-demographic variables and the top 10 correlates of wanting to seek help were presented in the S1 Table. The strongest interaction was found between current self-rated mental health and housing status (interaction strength 0.2). Other top 10 interactions involved intersections of housing status with sexual orientation, gender identities, past-year suicidal thoughts, average outness score and overall internalized homophobia score; interactions were found between sexual orientation and gender identities with self-rated mental health and past-year suicidal thoughts. Specifically, after stratified by mental health status, past-year suicidal thoughts, outness score and internalized homophobia score, individuals with adequate housing during the pandemic always had a higher probability of wanting to seek help than those experiencing inadequate housing (Fig 4). In addition, among young adults who self-identified themselves as bisexual, others, queer and questioning, gender diverse or cisgender, the risk of wanting to seek help was further increased by having adequate housing, poor or fair self-rated mental health or past-year suicidal thoughts (Figs 5 and 6).

### 3.5 Descriptive statistics and predicted risk of delay in accessing care

Of the total 1089 participants who wanted to seek help during the pandemic, 69.8% (n = 760) reported delay in accessing care and 25.8% (n = 281) had no delay in accessing care (Table 2). Participants who had delay in accessing care were slightly younger on average (mean age 21.63 ± 3.73 years, p = 0.007) and differed significantly (p = 0.002) by highest level of education completed. Particularly among those who had less than a university degree, 72.1% (n = 534) reported delay in accessing care. The RF model built for predicting risk of delay in accessing care failed to achieve satisfactory performance (AUC 0.61) on the testing data (S2 Appendix). The top 10 correlates were: average outness score (100%), overall internalized homophobia score (85.7%), overall ACEs score (81.8%), age (77.4%), overall connectedness score (77.3%), overall perceived stigma score (76.2%), overall enacted stigma score (73.4%), overall identity centrality score (71.2%), diagnosis of other mental illness (27.1%), current self-rated general health (26.4%) (S1 Fig). However, due to the unsatisfactory performance of this RF model, we deemed those findings to be less reliable.

**Table 2:** Socio-demographic characteristics of participants by delay in accessing care during the pandemic (n = 1089).

Characteristics	No delay in accessing care (n = 281)^a^	Delay in accessing care (n = 760)^b^	Total (n = 1089)^c^	p-value
**Age (years), mean (SD)**	22.34 (3.83)	21.63 (3.73)	21.75 (3.78)	0.007
**Sexual orientation, n (%)**	** **			0.582
Asexual	17 (34.0)	31 (62.0)	50 (4.6)	
Straight or heterosexual	1 (20.0)	3 (60.0)	5 (0.5)	
Bisexual	83 (24.5)	241 (71.1)	339 (31.1)	
Gay	37 (29.4)	85 (67.5)	126 (11.6)	
Lesbian	39 (23.1)	125 (74.0)	169 (15.5)	
Queer	52 (24.3)	148 (69.2)	214 (19.7)	
Questioning	2 (14.3)	11 (78.6)	14 (1.3)	
Others	50 (29.1)	116 (67.4)	172 (15.8)	
**Gender identity, n (%)**				0.461
Cisgender man	33 (28.4)	81 (69.8)	116 (11.2)	
Cisgender woman	114 (23.8)	348 (72.7)	479 (46.3)	
Transgender man	23 (29.1)	51 (64.6)	79 (7.6)	
Transgender woman	6 (35.3)	10 (58.8)	17 (1.6)	
Gender diverse	94 (27.3)	230 (66.9)	344 (33.2)	
**Intersex, n (%)**	5 (29.4)	12 (70.6)	17 (1.7)	1
**Ethnicity, n (%)**				0.580
White	187 (26.0)	496 (69.1)	718 (68.8)	
Person of colour	80 (24.5)	234 (71.8)	326 (31.2)	
**Education, n (%)**				0.002
High school or below	65 (19.5)	247 (74.2)	333 (30.9)	
College or diploma	107 (26.2)	287 (70.3)	408 (37.8)	
University degree	108 (32.0)	219 (65.0)	337 (31.3)	
**Employment, n (%)**				0.717
Unemployed	208 (26.3)	550 (69.4)	792 (73.0)	
Employed	73 (24.9)	207 (70.6)	293 (27.0)	
**Household income, n (%)**				0.62
Yearly less than $60,000	117 (25.5)	326 (71.2)	458 (56.2)	
Yearly $60,000- $100,000	46 (24.6)	135 (72.2)	187 (22.9)	
Yearly more than $100,000	50 (29.4)	118 (69.4)	170 (20.9)	
**Type of municipality, n (%)**				1
Rural	9 (25.7)	24 (68.6)	35 (3.2)	
Urban	272 (25.8)	736 (69.8)	1054 (96.8)	
**Housing, n (%)**				0.398
Inadequate	23 (21.9)	77 (73.3)	105 (9.7)	
Adequate	258 (26.4)	680 (69.5)	979 (90.3)	
**Substance use in past 12 months, n (%)**	97 (27.2)	245 (68.8)	356 (33.0)	0.491
**Anxiety disorder, n (%)**	157 (28.0)	375 (66.8)	561 (51.5)	0.072
**Depression, n (%)**	137 (28.0)	328 (67.1)	489 (44.9)	0.123
**General health, n (%)**				0.033
Poor or fair	105 (22.3)	345 (73.2)	471 (43.9)	
Good or excellent	170 (28.2)	407 (67.5)	603 (56.1)	
**Mental health, n (%)**				0.057
Poor or fair	208 (24.4)	603 (70.9)	851 (78.4)	
Good or excellent	73 (31.2)	153 (65.4)	234 (21.6)	
**Current stress level, n (%)**				0.178
Not stressful or neutral	98 (29.2)	230 (68.5)	336 (30.9)	
Very stressful or extremely stressful	183 (24.3)	530 (70.4)	753 (69.1)	
**COVID-19 impact on smoking, n (%)**				0.403
Don’t smoke	192 (26.0)	511 (69.1)	739 (67.9)	
Increased	32 (24.6)	91 (70.0)	130 (11.9)	
Decreased	16 (18.4)	68 (78.2)	87 (8.0)	
No change	29 (30.9)	64 (68.1)	94 (8.6)	
Quit	12 (30.8)	26 (66.7)	39 (3.6)	
**COVID-19 impact on alcohol and substance use, n (%)**				0.513
Don’t use	50 (29.6)	105 (62.1)	169 (15.5)	
Increased	104 (24.3)	313 (73.1)	428 (39.3)	
Decreased	56 (27.5)	143 (70.1)	204 (18.7)	
No change	55 (24.4)	155 (68.9)	225 (20.7)	
Quit	16 (25.4)	44 (69.8)	63 (5.8)	

Abbreviations: COVID-19, Coronavirus disease 2019; SD, Standard deviations.

^a^ The percentage in the brackets represents rate of the participants in each characteristics who had no delay in accessing care.

^b^ The percentage in the brackets represents rate of the participants in each characteristic who had delay in accessing care.

^c^ The percentage in the brackets represents percentage of the total participants in each characteristics.

Note: We used 2-sample t-test and Fisher’s Exact Test to compare the distribution of continuous and categorical variables between the two groups.

### 3.6 Sensitivity analysis

All alternative RF models built on the remaining 4 MICE-imputed copies of the datasets and with ‘missForest’ for wanting to seek help and delay in accessing care performed similarly with AUC ranging 0.82–0.85 and 0.56–0.61 respectively (S2 and S3 Appendix). For both outcomes, the model performance dropped considerably while the models were built on the completed cases datasets (AUC 0.75, AUC 0.52 respectively). The Lasso logistic regression model found 15 of the 61 predictors to have a non-zero coefficient when predicting the status of delay in accessing care, including 4 (average outness score, overall internalized homophobia score, self-rated general health, diagnosis of other mental illness) that were previously identified to be among the top 10 correlates by RF (S2 Appendix). However, this lasso regression model failed to show better performance on the testing set (AUC 0.57). Hence, we conclude that there is a lack of key predictors of delay in accessing care in this specific dataset.

## 4. Discussion

We applied machine learning to analyze factors associated with wanting to seek help for mental health and substance use concerns among 2SLGBTQI+ young adults during the pandemic. Using the resulted RF model that had moderately high performance, we identified correlates of wanting to seek help that depict vulnerable young adults in need of urgent interventions. Furthermore, we used the same RF technique to identify those who had delay in accessing care; however, this model demonstrated poor performance which impeded our ability to draw any conclusive findings on correlates and interactions.

The findings of our study support an emerging body of evidence [7,20,53] that a significant proportion of 2SLGBTQI+ young adults showed wanting to seek help during the pandemic, while most of them reported delay in accessing care (Tables 1 and 2). Moreover, we found that the rate of wanting to seek help was higher among those who decreased or quit their smoking, or increased drinking or substance use due to COVID-19 pandemic. These findings suggests that 2SLGBTQI+ young adults probably wanted services for quitting smoking or managing consequences of their substance use. The rate of wanting to seek help was significantly higher among unemployed young adults. However, it is important to recognise that most of the participants (80.6%, n = 741) in our sample did not finish a university degree and may be still studying at the undergraduate levels, which might be reflected by the higher rate of unemployment in the overall sample (Table 1).

The top 10 correlates of wanting to seek help among 2SLGBTQI+ young adults identified in our study were mainly related to mental health challenges, age, sexual and gender identity related stigma and discrimination, and ACEs (Figs 2 and 3). Consistent with previous research, we found an association between wanting to seek help and poor or fair self-rated mental health status [54,55]. Previous research reported decreased rate of seeking help among those who seriously considered or attempted suicide [54,56]. In our study, we found increased likelihood of wanting to seek help among participants who had suicidal thoughts in the past year. However, this particular finding of increased needs of seeking help among 2SLGBTQI+ young adults experiencing mental health challenges presents an opportunity for timely intervention. In addition to increased prevalence of mental health issues [6], making virtual mental health visits became more common during the pandemic [57]. Hence, 2SLGBTQI+ young adults can be encouraged to leverage these services, seek help and access urgent online supports at the time of mental health emergency.

The association of exposure to enacted stigma or discrimination, perceived stigma, homophobia, low community connectedness, low level of identity centrality with adverse mental health outcomes have been already demonstrated [22,58,59]. Our results shows that these experiences also increased risk of wanting to seek help. Previous research among 2SLGBTQI+ college students concluded that high level of outness was associated with high likelihood of affected by enacted stigma and high probability of help-seeking [60]. The same effect was demonstrated in our study for wanting to seek help. While more research are needed to explore ways to reduce stigma and discrimination against 2SLGBTQI+ people, tailored peer support programs can improve self-advocacy, community connectedness, and improve mental health as well as quality of life [61]. In addition, policy initiatives like anti-discrimination measures and LGBTQI2S+ inclusion in the socio-economic development [62] will help to reduce discrimination. Evidence suggests that high exposure to ACEs predicts poor mental health outcomes among SGM youth and adults [63,64]. Moreover, high ACEs score were found to be associated with ever-smoking and ever-vaping among the 2SLGBTQI+ young adults [61]. We found similar effect of increased likelihood of wanting to seek help among participants with high ACEs score. However, the association between wanting to seek help and low ACEs score more likely resulted from the data imputation of high percentage (11% of the total sample) of missing data for the ACEs questionnaire. While this relationship between ACEs and wanting to seek help should be further explored exclusively, the ACEs questionnaire can be used for screening mental health and substance use challenges among 2SLGBTQI+ young adults.

Our findings suggest that individuals who had adequate housing, but poor mental health or past-year suicidal thoughts were more likely to want to seek help than those with inadequate housing. However, a recent study reported that homeless 2SLGBTQI+ youth were experiencing poor mental health, suicidal thoughts, self-harm, increased substance use and other mental health issues as an impact of COVID-19 [20]. This difference may be attributable to numerous barriers (i.e., lack of affordability, lack of transportation, stigmatization) faced by homeless individuals which may discouraged them to seek healthcare [65]. Moreover, past experiences of stigmatization and discrimination from health care providers for their sexual and gender identities may further inhibit young people to seek help [9]. Consistent with this possibility, we found that participants with adequate housing and lower level of internalized homophobia or average outness had higher probability of wanting to seek help (Fig 4). Notably, in our sample, bisexual, queer and questioning, cisgender, and gender diverse participants, who experienced mental health challenges or had adequate housing, were more likely to want to seek help than others (Figs 5 and 6). This finding contradicts previous research, where trans people were similar to cisgender people or more likely to experience mental health challenges and seek help than cisgender participants [55,66]. While future research should explore the reason behind these differences, these findings should be taken into account for the purpose of targeted prevention and intervention to reduce health disparities faced by 2SLGBTQI+ young adults.

Both the RF model and the Lasso regression model built for predicting delay in accessing care had poor performance (S2 Appendix), largely attributed to our limited sample size and capture of key predictors. Notably, delay in accessing care result from a complex interplay of factors beyond the individual level (structures and processes of care), as outlined in the Donabedian model [67]. Nevertheless, a few individual-level variables (outness, internalized homophobia, self-rated general health, and other mental illness) were identified as potentially important correlates in both models, which may imply some meaningful mechanisms that underlie high risks of care delays. This aligns with research on real fears of violence, and social exclusion experienced by SGM people as well as lack of 2SLGBTQI+ friendly healthcare providers during disclosure [68]. Specifically, recent studies reported that SGM people faced significant rates of health care discrimination and harassments by healthcare providers, including refusal to provide care as well as inflicting verbal and physical abuse during the COVID-19 pandemic [7,9]. These experiences of discrimination within the healthcare system should be incorporated into future research to evaluate their impact on predicting the risk of delayed access to care. Future study with more extensive capture of variables is needed to identifying predictors of delay in accessing care by 2SLGBTQI+ young adults to enable policy planning that ameliorates health care discrimination and ensures health equity.

We demonstrated that machine learning can be used successfully to identify potential risk factors of mental health and substance use related issues among the 2SLGBTQI+ young adults, with an adequate sample size to capture of key predictors. Compared to traditional regression approaches, machine learning allowed us to investigate potentially complex relationships amongst 61 variables without imposing significant assumptions to identify statistically meaningful individual predictors as well as interactions [69,70]. This flexible data-driven technique is particularly useful in hypothesis-generating settings to support causality-testing investigations. As such, this study provides an example of deriving interpretable findings from a black-box machine learning model, which has been identified as being underperformed in the current public health literature [70,71]. Future public health researchers should be mindful of making their machine learning models interpretable and potentially use more sophisticated techniques (such as individual conditional expectation plots [72]) to enhance the real-world relevance of their interpretations. 2SLGBTQI+ people face various social and institutional barriers in accessing timely healthcare services, which are often intertwined with factors that challenge the disclosure of their identities [71]. By leveraging the data-driven strength of machine learning, future researchers could adopt our approach to uncover nuanced findings that hinder the help-seeking behaviours of this underserved population [11]. In addition, beyond supervised machine learning that we demonstrated in this study, techniques of unsupervised learning (cluster analysis [73]) can be applied to characterize unique preferences/trajectories of care that might be particularly suitable for this population to enable a patient-centred care approach.

The findings of this study should be interpreted with few key limitations. Although the RF model predicting status of wanting to seek help yielded relatively high performance, we were unable to achieve satisfactory performance for the models build for predicting delay in accessing care. This weakness of the current study was attributed to the inherent limitations of a cross-sectional survey-based study (e.g., small sample, missing responses, omission of potentially important attributes in the questionnaire). Future researchers should use more comprehensive datasets, preferably ones that link individual- and system-level factors from health services administrative databases with population-based survey data, to depict a fulsome picture of barriers to timely care receipt. Next, our primary outcome denoted the status of wanting to seek help, without confirming how many of them have actually subsequently sought (and received) professional help. This should be addressed in longitudinal studies. Furthermore, we were unable to comment on whether the participants started to experience the needs to seek help since the start of the pandemic or how the pandemic influenced changes in risk factors of wanting to seek help among 2SLGBTQI+ young adults. These unaddressed objectives can be explored in a case-control setting with data from the pre-pandemic era. Moreover, owing to the data driven nature of the machine learning techniques and cross-sectional data, the top 10 correlates identified were not ‘true predictors’, rather factors that might contribute to a larger increase in the likelihood of wanting to seek help than other variables. As such, the correlates suggested by this study should be confirmed using causality testing techniques to formally establish their statistical importance.

## 5. Conclusions

We conducted an explanatory machine learning analysis on Canadian 2SLGBTQI+ young adults during the COVID-19 pandemic to identify individual-level correlates of wanting to seek professional help and subsequent delay in accessing care. We identified several sexual and gender-identity related stressors influencing wanting to seek help, which should be taken into consideration by the policy makers for targeted prevention approaches and ensuring health equity. Interventions such as peer support programs, social inclusion of 2SLGBTQI+ people on socio-economic development, transportation and combined efforts for people experiencing homelessness, and 2SLGBTQI+ inclusive and affirming health care services should be considered for reducing mental health and substance use challenges as well as improving help-seeking among this population. The poor performance of the random forest models aimed to predicting delay in accessing care warrants additional research using more comprehensive datasets that include factors measured at structures and processes of care levels.

## Supporting information

S1 Fig The top ten correlates of delay in accessing care and their scaled relative importance. (TIFF)

S1 Table Interaction strengths formed by socio-demographic factors and top 10 important correlates (in descending order of interaction strength) of wanting to seek help. (DOCX)

S1 Appendix Analytical framework of the machine learning models. (DOCX)

S2 Appendix Results of sensitivity analysis for predicting risk of delay in accessing care. (DOCX)

S3 Appendix Results of sensitivity analysis for predicting risk of wanting to seek help. (DOCX)
