# Supplementary figures and images for: Correlates of wanting to seek help for mental health and substance use concerns by sexual and gender minority young adults during the COVID-19 pandemic: A machine learning analysis
Source: PLoS One. 2022 Nov 16;17(11):e0277438. doi: 10.1371/journal.pone.0277438 (PMC9668172; doi:10.1371/journal.pone.0277438)

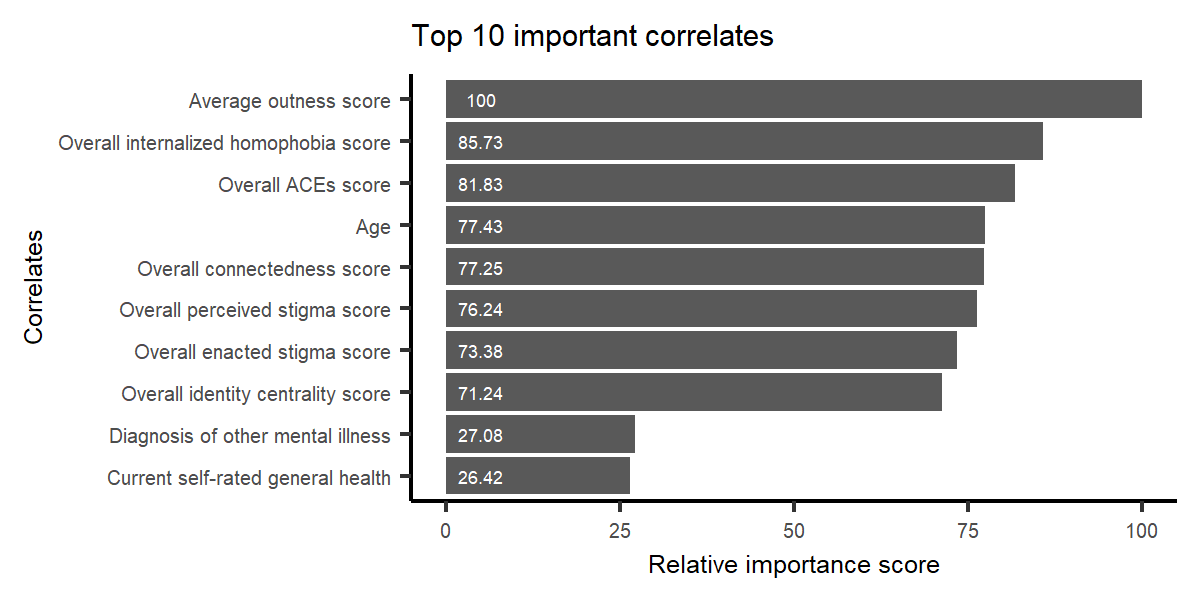

Supplement: S1 Fig — (TIFF) [file pone.0277438.s001.tiff]
