# Supplementary material for: Correlates of wanting to seek help for mental health and substance use concerns by sexual and gender minority young adults during the COVID-19 pandemic: A machine learning analysis
Source: PLoS One. 2022 Nov 16;17(11):e0277438. doi: 10.1371/journal.pone.0277438 (PMC9668172; doi:10.1371/journal.pone.0277438)
Supplement: S1 Table — (DOCX) [file pone.0277438.s002.docx]

**S1 Table. Interaction strength formed by socio-demographic factors and top 10 important correlates (in descending order of interaction strength) of wanting to seek help.**

| Variables | Interaction strength |
| --- | --- |
| where_live*mental_health | 0.2 |
| where_live*suicidal | 0.14 |
| curr_orient*mental_health | 0.13 |
| curr_orient*suicidal | 0.12 |
| curr_orient*where_live | 0.11 |
| gender*mental_health | 0.11 |
| where_live*outness | 0.11 |
| where_live*phobia | 0.11 |
| gender*suicidal | 0.1 |
| gender*where_live | 0.09 |
| education*mental_health | 0.09 |
| where_live*cen_identity | 0.09 |
| where_live*connect_com | 0.09 |
| age*where_live | 0.08 |
| where_live*per_stigma | 0.08 |
| where_live*ace | 0.08 |
| curr_orient*outness | 0.07 |
| curr_orient*phobia | 0.07 |
| education*suicidal | 0.07 |
| curr_orient*gender | 0.06 |
| age*curr_orient | 0.06 |
| curr_orient*cen_identity | 0.06 |
| curr_orient*connect_com | 0.06 |
| curr_orient*ace | 0.06 |
| house_income*mental_health | 0.06 |
| where_live*en_stigma | 0.06 |
| age*education | 0.05 |
| age*gender | 0.05 |
| curr_orient*education | 0.05 |
| house_income*where_live | 0.05 |
| education*where_live | 0.05 |
| gender*outness | 0.05 |
| gender*phobia | 0.05 |
| gender*per_stigma | 0.05 |
| employ*mental_health | 0.05 |
| gender*education | 0.04 |
| curr_orient*house_income | 0.04 |
| gender*house_income | 0.04 |
| curr_orient*en_stigma | 0.04 |
| curr_orient*per_stigma | 0.04 |
| gender*en_stigma | 0.04 |
| gender*cen_identity | 0.04 |
| gender*connect_com | 0.04 |
| gender*ace | 0.04 |
| education*outness | 0.04 |
| education*cen_identity | 0.04 |
| education*phobia | 0.04 |
| education*per_stigma | 0.04 |
| education*connect_com | 0.04 |
| education*ace | 0.04 |
| house_income*suicidal | 0.04 |
| house_income*ace | 0.04 |
| age*employ | 0.03 |
| curr_orient*employ | 0.03 |
| education*employ | 0.03 |
| age*house_income | 0.03 |
| gender*employ | 0.03 |
| education*en_stigma | 0.03 |
| employ*suicidal | 0.03 |
| employ*per_stigma | 0.03 |
| house_income*outness | 0.03 |
| house_income*cen_identity | 0.03 |
| house_income*phobia | 0.03 |
| house_income*connect_com | 0.03 |
| education*house_income | 0.02 |
| employ*where_live | 0.02 |
| ethnicity*mental_health | 0.02 |
| ethnicity*outness | 0.02 |
| employ*outness | 0.02 |
| employ*en_stigma | 0.02 |
| employ*cen_identity | 0.02 |
| employ*phobia | 0.02 |
| employ*ace | 0.02 |
| house_income*en_stigma | 0.02 |
| house_income*per_stigma | 0.02 |
| age*ethnicity | 0.01 |
| gender*ethnicity | 0.01 |
| ethnicity*where_live | 0.01 |
| ethnicity*education | 0.01 |
| ethnicity*house_income | 0.01 |
| ethnicity*suicidal | 0.01 |
| ethnicity*cen_identity | 0.01 |
| ethnicity*phobia | 0.01 |
| ethnicity*per_stigma | 0.01 |
| ethnicity*ace | 0.01 |
| employ*connect_com | 0.01 |
| curr_orient*ethnicity | 0 |
| employ*house_income | 0 |
| ethnicity*employ | 0 |
| ethnicity*en_stigma | 0 |
| ethnicity*connect_com | 0 |
