# Supplementary material for: Correlates of wanting to seek help for mental health and substance use concerns by sexual and gender minority young adults during the COVID-19 pandemic: A machine learning analysis
Source: PLoS One. 2022 Nov 16;17(11):e0277438. doi: 10.1371/journal.pone.0277438 (PMC9668172; doi:10.1371/journal.pone.0277438)
Supplement: S1 Appendix — (DOCX) [file pone.0277438.s003.docx]

**S1 Appendix: Analytical Framework of the machine learning models**

Graphical representation of the analytical framework:


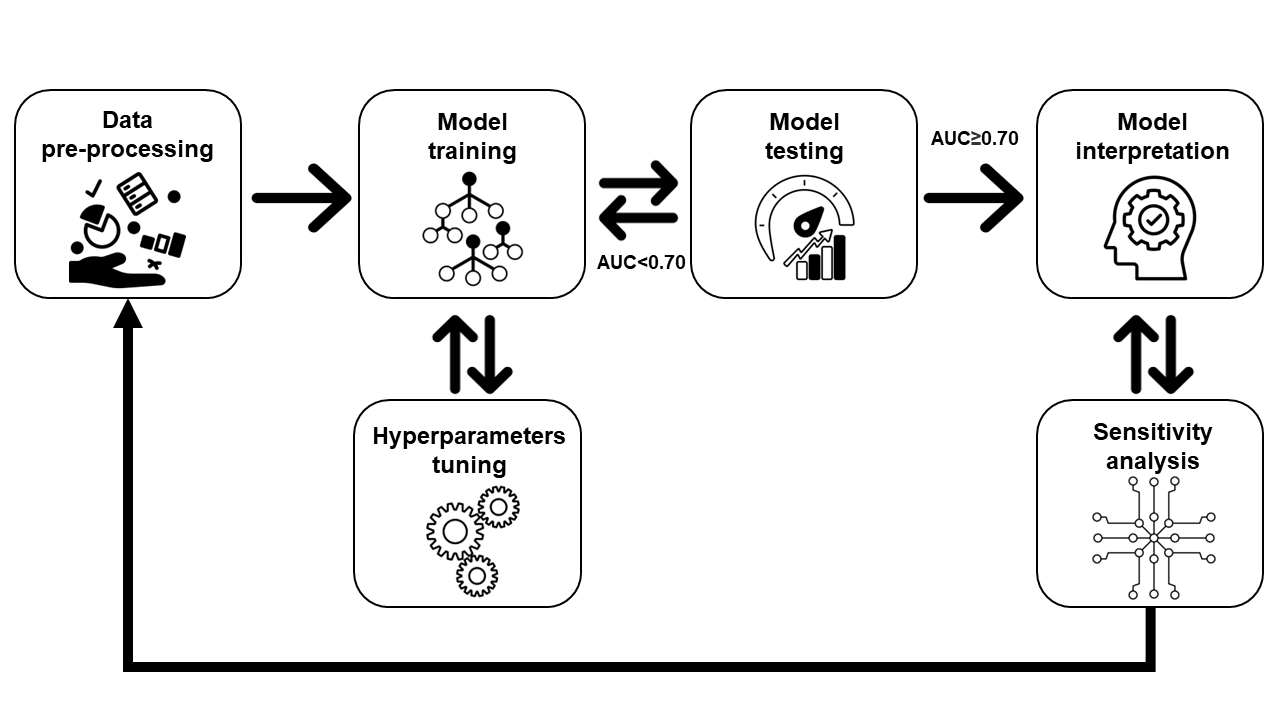


**Data preprocessing:**

1. Remove/recoded low-variance variables
2. Ensured an absence of collinearity amongst features (Cramer’s V<0.6)
3. Inspect missing data (total missingness=2.8%). We used Multivariate Imputation by Chained Equations (MICE) to generate 5 complete data sets. Features that were continuous, binary, or categorical with > 2 levels were predicted via predictive mean matching, logistic regression, or a polytomous regression model, respectively. We reported the MICE-imputed dataset leading to the best-performing model (highest AUC) in the primary analysis and reserved the description of the remaining 4 models in the sensitivity analysis.

Visual inspection of missing values in the dataset (column=variable, row=observation):


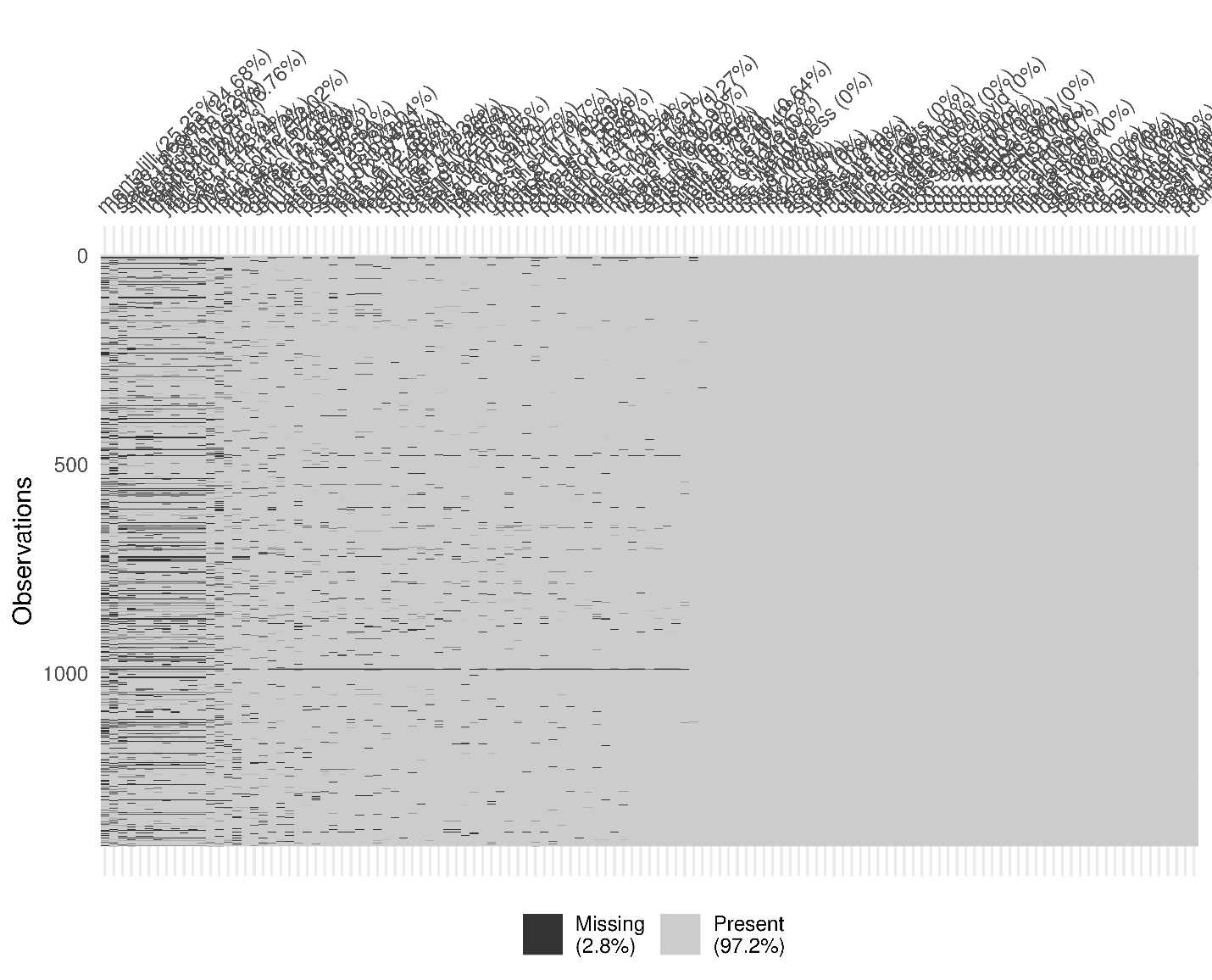


**Model training**: we developed a random forest algorithm and a Lasso logistic regression algorithm (if the random forest has poor performance, i.e., AUC≤0.70).

*Random forest:*

1. Randomly split the dataset into training and testing using a ratio of 70:30.
2. Develop a random forest using default hyperparameters and 10-fold cross-validation:
   1. number of trees=500
   2. number of variables tried at each binary split=square root of number of predictor variables=8 (61 variables)

*Logistic lasso regression:*

The basic model setup is as followed:

$$P\left( Y=1 | x_{i} \right)=\frac{exp(\beta_{0}+\beta_{1}x_{i1}+\ldots+\beta_{k}x_{ik})}{1+exp(\beta_{0}+\beta_{1}x_{i1}+\ldots+\beta_{k}x_{ik})}$$

where P(Y=1|X) denotes the probability of a positive outcome (i.e., having wanted to seek help or having experienced delays in care). Our goal was to estimate parameters $\hat{\boldsymbol{\beta}}$**.**

The loss function of this logistic regression model is the negative log-likelihood function:

$$L=\sum_{i=1}^{n} [-y_{i}(\beta_{0}+\beta_{1}x_{i1}+\ldots+\beta_{k}x_{ik})+\log\left( 1+\exp\left( \beta_{0}+\beta_{1}x_{i1}+\ldots+\beta_{k}x_{ik} \right)) \right]$$

In a 10-fold cross-validation process, we find optimal $\lambda>0$ that minimizes L subject to the penalty term:

$$\lambda\sum_{j=1}^{k} |\beta_{j}|$$

As such, for unimportant variables $x_{i}$, its coefficient $\hat{\beta_{j}}$ will be shrank to 0, thereby achieving a sparse model.

**Model testing:** we computed the following statistics to evaluate the performance of the trained model on a testing dataset. Decision threshold was fixed at 50%.

$$Sensitivity=\frac{TP}{TP+FN}$$

$$Specificity=\frac{TN}{TN+FP}$$

$$Accuracy=\frac{TP+TN}{TP+FN+TN+FP}$$

The area under a receiver operating characteristic curve (AUC) was also computed where perfect classifier = 1.0 and flip of coin = 0.50. We used < 0.70 as a threshold to declare inadequate performance.

**Model interpretation:**

*Variable importance ranking*

We used permutation importance to rank the top ten most important contributing features in the random forest.

1. For each classification tree, on the out-of-bag sample, the value of the variable of interest was randomly shuffled, while keeping the other variables at constant.
2. The decrease in prediction accuracy on the shuffled data was measured.
3. The total decrease in accuracy in the forest was computed and divided by the number of trees to yield mean decrease in accuracy due to omission/shuffling of this variable.
4. We standardized the mean decrease in accuracy between 0-100% so the most important variable got 100%.

*Interaction strength*

We used partial dependence based 2-way interaction effects by Greenwall et al., 2018 to examine interactions between pairs of variables formed by sociodemographic variables (such as age*gender) and/or the identified top 10 correlates of wanting to seek help.

It measures feature interaction between feature *A* and feature *B* with:

S*_A_* = *sd*(i(*A*)|*B*j)

S*_B_* = *sd*(i(*B*)|*A*j)

Interact(*A,B*) = (S*_A_* + S*_B_*)/2

Where *sd* is standard deviation; i(*A*) is feature importance of *A*; j = 1, 2, 3, . . . , *n*; and *A_i_* is an *i*-th feature value of A.

The interaction strengths of total 91 pairs ranged from 0 to 0.2 (see S1 Table). Out of 91 pairs top 10 pairs with the highest interaction strengths were selected to present by 2-way partial dependence plots.

**Sensitivity analysis:**

1. We reported the model performance/interpretation findings using the remaining 4 MICE-imputed data sets.
2. We applied the ‘missForest’ method to impute the missing data and to train another random forest algorithm simultaneously:
3. Randomly split the unimputed dataset into training and testing using a ratio of 70:30.
4. MissForest was applied separately on the training and testing set to impute the missing data. The imputation starts with replacing the missing values by mean of the observations for variables X*_s_, s*={1,….,*p*}. The missing values imputation for each variable X*_s_* continues by fitting random forest on the predictors X^(s)^_obs_ and the outcome Y^(s)^_obs_ and applying it to predict Y^(s)^_miss_ from X^(s)^_miss_. This procedure repeats until the stopping point criterion holds, which is when the difference between the new and old imputed values increases for the first time. The difference for set of continuous variables N is:


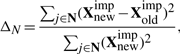


For the set of categorical variables F is:


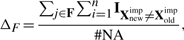


1. RF model was built on the imputed training set.
2. Trained model performance was determined on the imputed testing set.
3. We repeated the random forest analysis on a complete-case data set.
4. We conducted Lasso on the primary MICE imputed dataset for the delay in accessing care outcome.
