# Supplementary material for: Correlates of wanting to seek help for mental health and substance use concerns by sexual and gender minority young adults during the COVID-19 pandemic: A machine learning analysis
Source: PLoS One. 2022 Nov 16;17(11):e0277438. doi: 10.1371/journal.pone.0277438 (PMC9668172; doi:10.1371/journal.pone.0277438)
Supplement: S2 Appendix — (DOCX) [file pone.0277438.s004.docx]

**S2 Appendix: Sensitivity analysis for the subgroup analysis of delay in accessing care.**

Parameters of different random forest (RF) models built for predicting risk of delay in accessing care:

|  | AUC | Accuracy  (95% CI) | Sensitivity  (95% CI) | Specificity  (95% CI) |
| --- | --- | --- | --- | --- |
| Imputed dataset 1  (primary dataset) | 0.61 | 0.73 (0.68, 0.78) | 0.00 (0.00, 0.04) | 1.00 (0.98, 1.00) |
| Imputed dataset 2 | 0.57 | 0.73 (0.68, 0.78) | 0.00 (0.00, 0.04) | 1.00 (0.98, 1.00) |
| Imputed dataset 3 | 0.58 | 0.73 (0.68, 0.77) | 0.00 (0.00, 0.04) | 1.00 (0.98, 1.00) |
| Imputed dataset 4 | 0.60 | 0.72 (0.66, 0.76) | 0.07 (0.03, 0.14) | 0.95 (0.91, 0.97) |
| Imputed dataset 5 | 0.60 | 0.73 (0.68, 0.78) | 0.00 (0.00, 0.04) | 1.00 (0.98, 1.00) |
| Model built with ‘missForest’ | 0.56 | 0.74 (0.69, 0.79) | 0.00 (0.00, 0.04) | 1.00 (0.98, 1.00) |
| Completed cases (unimputed dataset) | 0.52 | 0.72 (0.62, 0.81) | 0.00 (0.00, 0.14) | 1.00 (0.94, 1.00) |

*Final model RF parameters:*

AUC: 0.606

Accuracy: 0.73(95% CI 0.68, 0.78)

Sensitivity: 0.00 (95% CI 0.00, 0.04)

Specificity: 1.00 (95% CI 0.98, 1.00)

Positive predictive value: NaN (95% CI 0.00, 1.00)

Negative predictive value: 0.73 (95% CI 0.68, 0.78)

ROC Curves of all random forest models and lasso regression for predicting risk of delay in accessing care:

**
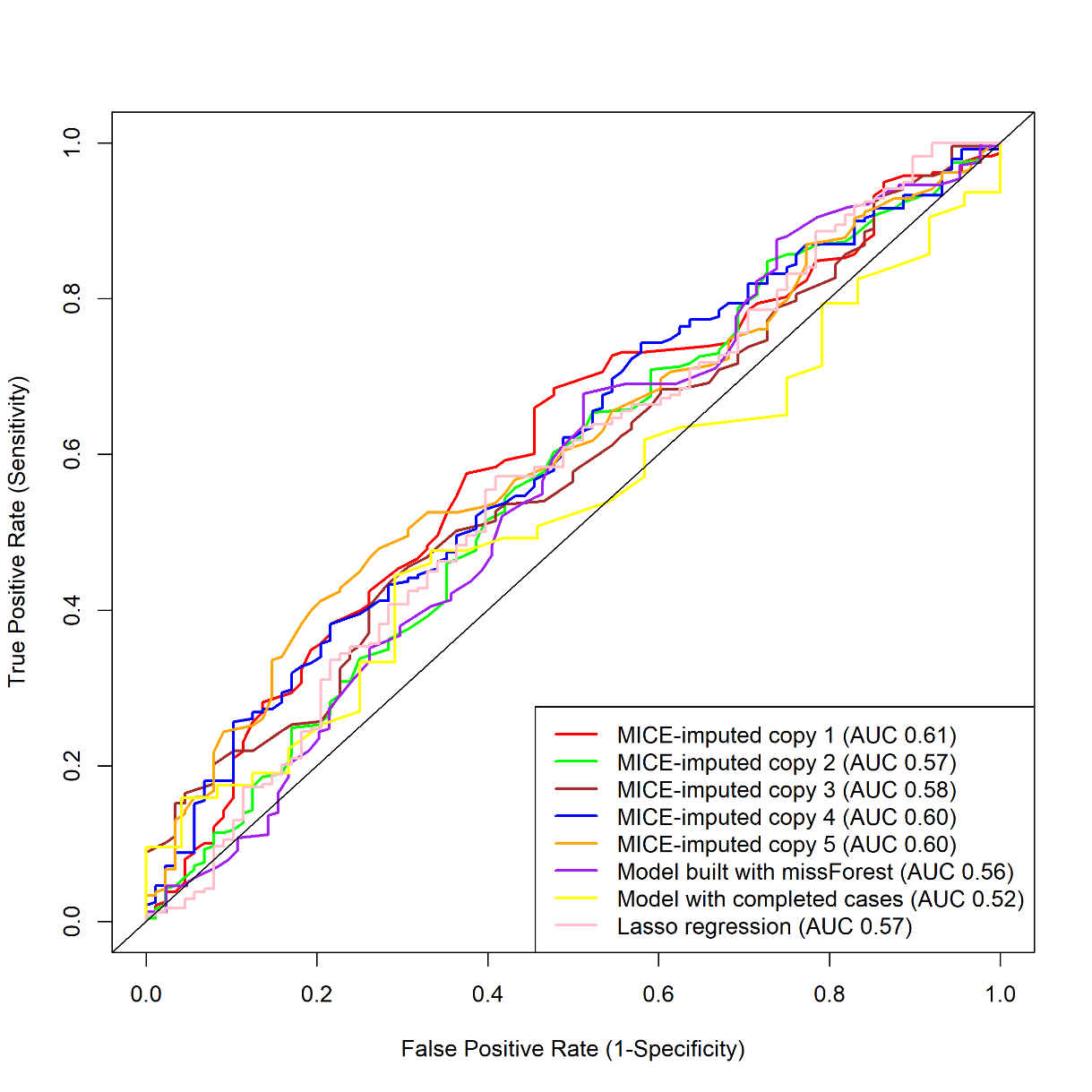
**

Co-efficients of variables following Lasso logistic regression:

| Variable codename | Co-efficient |
| --- | --- |
| curr_smoke1 | 0 |
| curr_smoke2 | 0 |
| curr_smoke3 | 0 |
| use_cigar1 | 0 |
| use_wp1 | 0 |
| use_smokeless1 | 0 |
| covid1 | 0 |
| covid2 | 0 |
| covid3 | 0 |
| risk12 | 0 |
| risk13 | 0.064063 |
| risk14 | 0 |
| risk15 | 0 |
| risk21 | -0.25794 |
| risk22 | 0 |
| risk31 | 0 |
| risk32 | 0 |
| risk41 | 0 |
| risk42 | 0 |
| rates1 | 0 |
| rates2 | 0 |
| accept1 | 0 |
| accept2 | 0 |
| stress1 | 0 |
| stress2 | 0 |
| stigma1 | 0 |
| stigma2 | 0.145784 |
| pressure1 | 0.136112 |
| pressure2 | 0 |
| mhealth1 | 0 |
| mhealth2 | 0.010863 |
| culture1 | 0 |
| culture2 | 0 |
| quit_attempts1 | 0 |
| quit_attempts2 | 0 |
| tailored1 | 0 |
| tailored2 | 0 |
| quit_support1 | 0 |
| quit_support2 | 0 |
| quit_support3 | 0 |
| curr_vape1 | 0 |
| curr_vape2 | 0 |
| alcohol_amount1 | 0 |
| alcohol_amount2 | 0 |
| cannabis1 | 0 |
| cannabis2 | 0 |
| substances_covid1 | 0 |
| substances_covid2 | 0 |
| substances_covid3 | 0 |
| gen_health1 | -0.27942 |
| fitness11 | -0.04983 |
| mental_health1 | 0 |
| stresslife1 | 0 |
| con_eating1 | 0 |
| con_anxiety1 | -0.03923 |
| con_ADD1 | 0 |
| con_ADHD1 | 0 |
| con_biploar1 | 0 |
| con_depression1 | -0.01164 |
| con_OCD1 | 0 |
| con_panic1 | 0 |
| con_PTSD1 | 0 |
| con_others1 | -0.3385 |
| suicidal1 | 0 |
| residence1 | 0 |
| education1 | 0 |
| education2 | 0 |
| house_income1 | 0 |
| house_income2 | -0.00804 |
| ind_income1 | 0 |
| ind_income2 | 0 |
| where_live1 | 0 |
| age | -0.0446 |
| province9 | 0 |
| curr_orient1 | 0 |
| curr_orient2 | 0 |
| curr_orient3 | 0 |
| curr_orient4 | 0 |
| gender1 | -0.07911 |
| gender2 | 0 |
| ethnicity1 | 0 |
| drug_12m1 | 0 |
| treat_comorbid1 | 0 |
| disability1 | 0 |
| employ1 | 0 |
| depress_score1 | 0 |
| depress_score2 | 0.083653 |
| cen_identity | 0 |
| outness | -0.04619 |
| connect_com | 0 |
| per_stigma | 0 |
| en_stigma | 0 |
| phobia | 0.031909 |
| ace | 0 |
