# Supplementary material for: Correlates of wanting to seek help for mental health and substance use concerns by sexual and gender minority young adults during the COVID-19 pandemic: A machine learning analysis
Source: PLoS One. 2022 Nov 16;17(11):e0277438. doi: 10.1371/journal.pone.0277438 (PMC9668172; doi:10.1371/journal.pone.0277438)

**S3 Appendix: Results of sensitivity analysis for predicting risk of wanting to seek help.**

Parameters of different random forest (RF) models built for predicting risk of wanting to seek help:

|  | AUC | Accuracy (95% CI) | Sensitivity (95% CI) | Specificity (95% CI) |
| --- | --- | --- | --- | --- |
| Imputed dataset 1 | 0.82 | 0.87 (0.83, 0.90) | 0.48 (0.37, 0.59) | 0.97 (0.95, 0.99) |
| Imputed dataset 2 | 0.84 | 0.87 (0.83, 0.90) | 0.49 (0.38, 0.60) | 0.97 (0.95, 0.99) |
| Imputed dataset 3 | 0.83 | 0.86 (0.83, 0.89) | 0.49 (0.39, 0.60) | 0.96 (0.93, 0.98) |
| Imputed dataset 4  (primary dataset) | 0.85 | 0.86 (0.83, 0.89) | 0.53 (0.42, 0.63) | 0.95 (0.92, 0.97) |
| Imputed dataset 5 | 0.82 | 0.87 (0.83, 0.90) | 0.50 (0.39, 0.61) | 0.97 (0.95, 0.99) |
| Model built with missForest | 0.83 | 0.87 (0.83, 0.90) | 0.48 (0.37, 0.59) | 0.97 (0.94, 0.98) |
| Completed cases (unimputed dataset) | 0.75 | 0.80 (0.71, 0.87) | 0.00 (0.00, 0.18) | 0.97 (0.91, 0.99) |

*Final model RF parameters:*

AUC: 0.85

Accuracy: 0.86 (95% CI 0.83, 0.89)

Sensitivity: 0.53 (95% CI 0.42, 0.63)

Specificity: 0.95 (95% CI 0.92, 0.97)

Positive predictive value: 0.75 (95% CI 0.62, 0.85)

Negative predictive value: 0.88 (95% CI 0.85, 0.91)

Receiver operating characteristic (ROC) Curves of all random forest models for predicting risk of wanting to seek help:


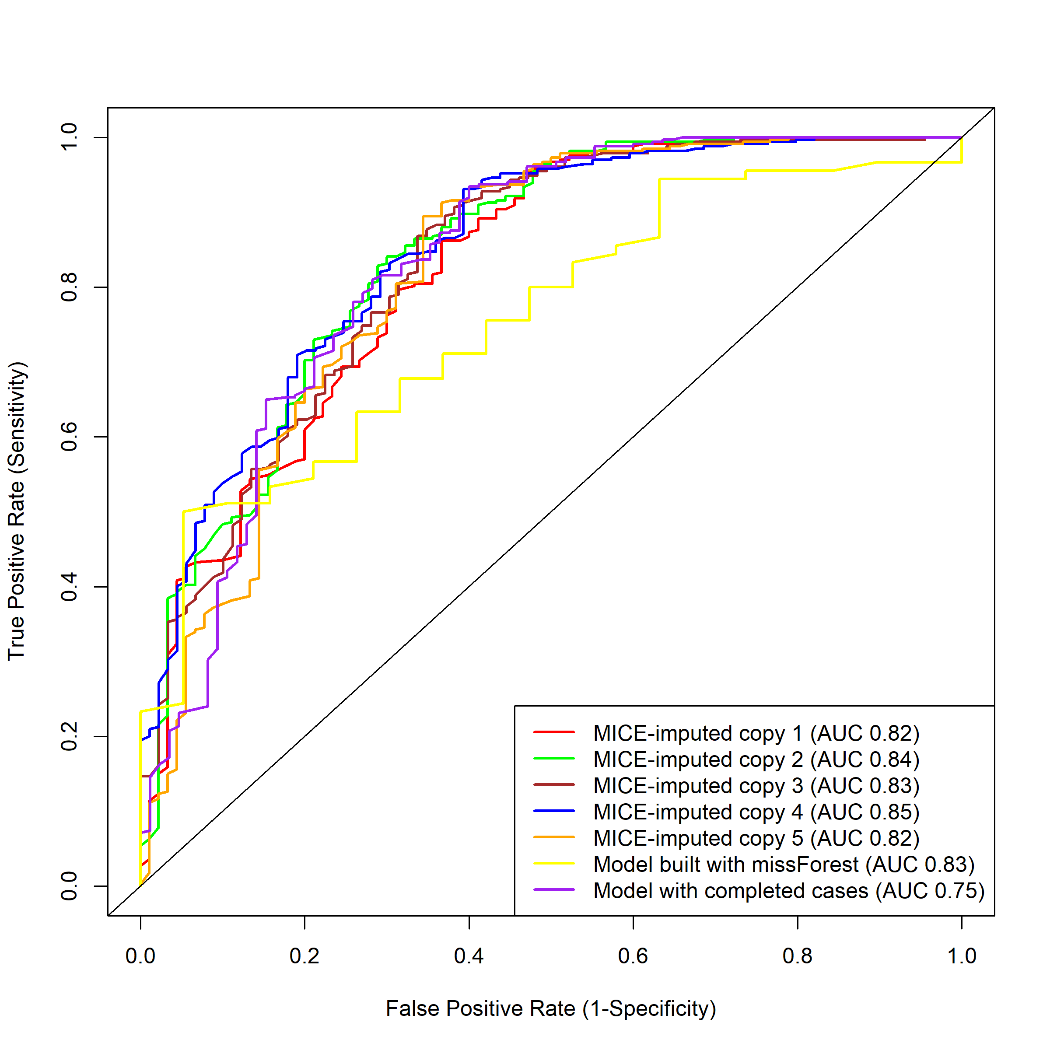

Supplement: S3 Appendix — (DOCX) [file pone.0277438.s005.docx]
